# Returning to work after sickness absence due to common mental disorders: study design and baseline findings from an 18 months mixed methods follow-up study in Germany

**DOI:** 10.1186/s12889-019-7999-z

**Published:** 2019-12-10

**Authors:** Alexandra Sikora, Gundolf Schneider, Ralf Stegmann, Uta Wegewitz

**Affiliations:** Federal Institute for Occupational Safety and Health (BAuA), Nöldnerstr. 40-42, 10317 Berlin, Germany

**Keywords:** Return to work, Common mental disorders, Prospective cohort study, Psychiatric treatment, Medical rehabilitation, Narrative interviews, Sample description, Work accommodation needs

## Abstract

**Background:**

With nearly 30 % of the general population experiencing one mental disorder in 12 months, common mental disorders (CMDs) are highly prevalent in Germany and mainly affect the workforce. Therefore, the processes of successfully returning to work (RTW) and achieving a sustainable RTW (SRTW) are important not only for recovery but the prevention of negative consequences like job loss or disability retirement. While factors influencing and predicting the time until RTW are well-investigated in other countries, research on determinants of RTW and SRTW has received little attention in Germany. Consequently, this study aims to investigate the RTW and SRTW processes due to CMDs from the employees´ perspective in Germany.

**Methods:**

This prospective cohort study uses a convergent parallel mixed methods design with a quantitative sample and qualitative sub-sample. Two hundred eighty-six participants of the quantitative study and a sub-sample of 32 participants of the qualitative study were included. The primary outcome of the quantitative study is the time until RTW and full RTW. The secondary outcome is the sustainability of RTW. The following measures will be used to cover work-, RTW- and health-related factors: working time, duration of sickness absences, functional ability, work ability, RTW self-efficacy, social support, work-privacy conflict, job satisfaction, job crafting and depressive symptoms. Quantitative and qualitative data will be integrated at the end.

**Discussion:**

The paper provides an overview on study design, recruitment, sample characteristics and baseline findings of an 18 months mixed methods follow-up study in Germany. This study will provide evidence of (S)RTW processes and its influencing factors due to CMDs in Germany and therefore contribute to further improvement of its (S)RTW practices.

**Trial registration:**

German Clinical Trials Register (ID: DRKS00010903, July 28, 2017, retrospectively registered).

## Background

Mental disorders are widespread in Germany. Twenty-eight percent of the general population between 18 and 79 years experience one mental disorder within a 12 month time period. The highest prevalence rates are observed among younger age groups, mainly affecting the working age (18–34 years: 36%, 35–49 years: 28%, 50–64 years: 26%, 65–79 years: 20%) [1, 2]. The burden, costs and challenges of mental disorders are high for the affected individuals, their employers, companies and society [3–6]. The processes of successfully returning to work (RTW) and achieving a sustainable RTW (SRTW), therefore, play an essential role in recovery and the prevention of further negative consequences like job loss or disability retirement [7–10].

Germany has a mainly community-based comprehensive mental healthcare system without great financial barriers for its use by the patients. Although the prevalence rates of mental disorders are high, utilisation rates of mental health services in Germany are relatively low, e.g. 33% with any 12-month diagnosis of a mood disorder report a mental health service use within the last 12 months, throughout all diagnoses the number is even lower (19%) [11]. Along with it, the German mental healthcare system is fragmented between various service providers (e.g. general practitioners vs. mental health specialists like psychiatrists or medical / psychological psychotherapists), settings (e.g. in- vs. outpatient care) and funding (e.g. statutory health insurances vs. German Pension Insurance scheme) [12, 13]; for a short overview of sectors and service providers see [11]. Despite the huge burden and costs, RTW and SRTW research at the intersection of the mental healthcare system and workplace has received little attention in Germany so far.

Previous research regarding RTW due to common mental disorders (CMDs) in other countries, especially cohort studies in the Scandinavian countries and the Netherlands [14, 15], has been primarily focused on factors influencing and predicting the time until RTW [14–16]. The literature has examined certain individual and external factors that are linked to a shorter time to RTW: Personal facilitators for RTW are higher self-efficacy (RTW-SE), positive RTW expectations, higher work ability and younger age. Disease-related factors are, in particular, the severity of the symptoms and the duration of the sick leave. Work-related factors relate mainly to the social support offered by the line managers and colleagues, as well as the collaboration and communication with employers during sickness absence; other facilitators are a gradual RTW (GRTW), the realisation of job accommodations and the timing of RTW [14–17].

However, much of this research concentrated particularly on the process towards RTW after suffering a mental disorder [18, 19]. According to the conceptualisation of RTW by Young et al. [20], in which RTW is seen as developmental process, two important phases after RTW (‘maintenance’ and ‘advancement’) have often been missing. In addition, as Nielsen et al. [18] pointed out, research that focuses on sustainable RTW (SRTW), investigating work-related measures and resources post-RTW (on individual, group, leadership and organisational levels which help to prevent relapse) is still insufficient, which makes it difficult to give valid recommendations for implementing and supporting SRTW [21]. Another limitation of previous RTW research due to CMDs is its focus on determinants, mostly disregarding research on workplace accommodations and needs for people with mental disorders. A scoping review [22] indicates that such workplace accommodations are not yet fully understood and that future studies should address which workplace accommodations are needed and how they can be realised.

Therefore, the present study was established to provide evidence from Germany regarding determinants of RTW and SRTW within its specific social security system. In addition, work accommodation needs and realised work accommodations during RTW will be considered. Finally, as the RTW and SRTW processes with their interactions are very complex, a mixed methods approach will be used to gain more detailed insights and understandings for the further improvement of RTW and SRTW practices in Germany.

### Aims

The overall aim of this mixed methods follow-up study is to investigate the RTW and SRTW processes at the intersection of the mental healthcare system and the workplace due to CMDs from the employees´ perspective in Germany. The following research aims are addressed in the quantitative study: (1) Identification of determining personal, disease- and work-related factors influencing the time until RTW and SRTW; (2) Description of employees´ RTW and SRTW trajectories and analysis of their relationship with personal, disease- and work-related factors. Research aims of the qualitative study are (1) to illustrate the interaction between personal factors, previous experiences, clinical treatment and work-related factors regarding RTW; (2) Reconstructing and describing the employees´ experiences, behaviours and actions, their implicit knowledge and frameworks for action during RTW. At the end, quantitative and qualitative data analyses will be integrated.

The purpose of this paper is to describe the study design, the recruitment process and the quantitative and qualitative baseline findings with its sample characteristics, particularly taking into account the differences between the treatment settings.

## Methods

### Jurisdictional context

In Germany, employees on sick leave receive full payment from their employer during the first 6 weeks of an illness every year. In small companies with up to 30 employees, employers can get a partial refund for these payments [23]. After 6 weeks of sickness absence, employees receive sickness absence benefits from their (statutory) health insurance (up to 78 weeks per illness every 3 years and about 70% of the full payment [24]). Employees have to hand over a medical certificate from their treating physician to their employer by the third day of absence from work, though a company can demand a medical certificate already on the first day of absence from work. The employer receives no information regarding the employees´ diagnosis and it is not relevant for the payment of sickness absence benefits if the illness was work-related or not.

If a sick leave period lasts longer than 6 weeks, all employers in Germany are legally responsible to offer their employees support to overcome work incapacity, to return to work and to prevent further sickness absences as well as disability retirement. This workplace integration management process has been regulated by law since 2004 and is called *Betriebliches Eingliederungsmanagement* (BEM). Despite the law regulating the setting, i.e. it is an obligation for the employer to offer BEM to the employees, it is not regulated how the process is realised (in each company) and which concrete measures are offered and agreed on by the employee and employer.

One specific strategy to facilitate RTW after long-term sickness absence is ‘gradual return to work’ (GRTW) [25–27]. In Germany, before a GRTW is initiated, physicians, mainly general practitioners (GPs), decide together with the employee which tasks he or she can perform and to what extent. During GRTW, employees are still certified as sick and receive sickness absence benefits, normally provided by their health insurance – or, within 4 weeks after medical rehabilitation, by the German Pension Insurance [28].

### Study design

The present mixed methods follow-up study addresses the processes of RTW and SRTW after sickness absence due to CMDs (depressive disorders, anxiety disorders and adjustment disorders) from the employees´ perspective in Germany. Within this prospective cohort study a convergent parallel mixed methods design is used. The quantitative and qualitative data were collected independently and in parallel. They will be analysed separately and the results will be integrated at the end [29].

### Recruitment and participants

Although the majority of people with mental disorders in Germany is treated in an outpatient setting [30], the study team decided in terms of the outcome of interest, narrow inclusion criteria and feasibility due to the fragmented mental healthcare system to only recruit participants from an inpatient treatment setting. Psychiatric clinics and rehabilitation facilities belong to the main mental health services for the prevention, cure, rehabilitation and continuing care in Germany [31] and were, therefore, further considered. Regarding depression, most acute inpatient services in Germany are provided by psychiatric departments [13]. Hence, psychiatric hospitals with inpatient and day programs were chosen to provide one part of the study group. Medical psychosomatic rehabilitation was chosen to provide the other part of the study group, because it took on greater significance during the last decades in consequence of the enormous increase of disability pensions due to mental disorders in Germany and is also mainly provided as inpatient treatment [32].

To gain access to the target group, the project team cooperated with two psychiatric clinics and three medical psychosomatic rehabilitation facilities. Psychiatric clinics provide help with psychotherapeutic, psychopharmacological and somatic program elements in acute crisis situations as a first responder or via referral through a registered physician by prior appointment. Furthermore, they often provide additional day hospital programs, where patients go home in the evenings and on weekends. The incurred costs are normally paid for by the health insurer.

Whereas acute psychiatric care has its focus on curative treatment, medical rehabilitation facilities provide help `to improve work ability and prevent disability pensions´ (p. 1, Bethge et al. [33]). Hence, medical rehabilitation has its focus on overcoming functional impairments to increase work and everyday life capacity [32]. A stay in a rehabilitation facility can either follow a psychiatric treatment, or, if a psychiatric treatment is not appropriate (e.g. due to less acute or severe symptoms), one can claim benefits for rehabilitation from the German Pension Insurance, where the need for rehabilitation is checked. If rehabilitation treatment is granted, costs are normally taken on by the German Pension Insurance. Psychiatrists and psychotherapists in an ambulant setting or GPs often initiate a rehabilitation treatment when preventive or acute measures were not successful enough. During a stay in a psychosomatic rehabilitation facility, a psychiatrist or medical psychotherapist has the main responsibility for the entire care process [34, 35].

Patients from the cooperating clinics were eligible for the study when they fulfilled the following criteria: (1) aged between 18 and 60 years, (2) sick-listed for less than 6 months during the last 12 months, (3) part-time (at least 15 h per week) or full-time employment, (4) permanent employment or fixed-term employment for at least 18 months, (5) intending to return to work with the former company and (6) treated for a first diagnosis and no more than one further diagnosis of the following list of disorders: a) depressive episode (F32.0, F32.1, F32.2), b) recurrent depressive disorder (F33.0, F33.1, F33.2), c) agoraphobia with panic disorder (F40.01), d) panic disorder (F41.0), e) generalized anxiety disorder (F41.1), f) mixed anxiety and depressive disorder (F41.2), g) adjustment disorders (F43.2). Patients with other disorders not mentioned under ‘Inclusion Criteria’, any current severe somatic comorbidity, minor employment, further qualification, unemployment or early retirement, without any intention to return to work (e.g. to apply for unemployment benefits) or insufficient knowledge of the German language to participate in a telephone interview were excluded.

Cooperating clinicians were briefed on the inclusion criteria and they subsequently recruited potential participants from August 2016 until November 2017. After obtaining approval and informed consent from the interested participants, a short paper-pencil questionnaire regarding the qualifying inclusion criteria, documents including the medical diagnoses and the participants’ date of discharge were delivered to the project team.

### Data collection and measurements

**Fig. 1:**
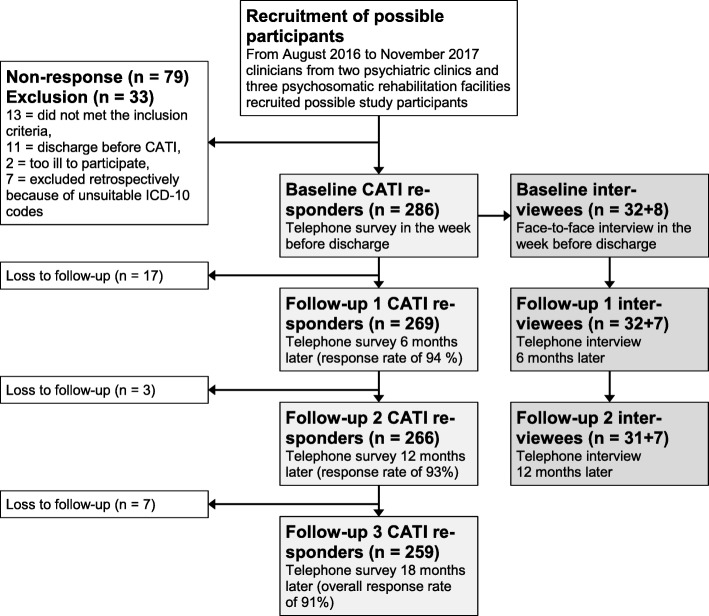
Flow of participants

It was planned that 300 participants should take part in four telephone surveys (quantitative study) and a sub-sample of 32 participants should take part in the interviews (qualitative study), see Fig. 1 for the study procedure. Sample size calculation was done by using an unadjusted regression model (log rank test of survival in two groups followed for fixed time, constant hazard ratio; α = .05; 2-sided test; hazard ratio = 0.7; power = 0.80). To ensure adequate statistical power, a total number of *n* = 247 events is required, but it must be assumed that with an adjustment of confounding variables, error variance will be decreasing and therefore power will be further increasing. Moreover, the sample size was deemed appropriate considering a response rate of 85% in comparable study designs, leading to a complete follow-up cohort of 255 participants with four measurements. The sub-sample for the qualitative interviews was selected by 1) additional qualitative approval and informed consent, 2) sex (16 women and 16 men), 3) RTW expectations from the short paper-pencil questionnaire (75% with a positive RTW expectation and 25% with a negative RTW expectation) and 4) first diagnosis from the clinicians (16 participants with depression, six participants with anxiety disorder and six participants with adjustment disorder). The quantitative study team selected the participants for the qualitative sub-sample from the main sample, giving the qualitative study team no information regarding the referred criteria, so that the qualitative study team was as unbiased as possible prior to the narrative interviews.

### Quantitative study

Computer-assisted telephone interviewing (CATI) was used on an offline computer to ensure a high level of data protection (offline version of LimeSurvey 2.50+). The participants of the quantitative study were questioned via telephone at four measurement points (at the end of the clinical treatment (t0), after 6 months (t1), 12 months (t2) and 18 months (t3), see Fig. 1). Baseline data collection took place between August 2016 and November 2017. Each telephone survey took 30 min on average. Participants received an immediate expense allowance of 25 Euro after completing each survey.

#### Quantitative measurements

Table 1 presents the selected questionnaire measures, their possible range (if appropriate) and measurement points. Baseline measurement included the following questions about socio-demographic and job characteristics: sex, age, education, partnership, cohabitation, monthly net household income, occupation, weekly working time, working time model, sedentary work, managerial function, tenure, enterprise sector and company size. Two demographic characteristic questions were asked at t1: type of health insurance (statutory or private) and “Are you a civil servant (yes or no)?”. All three follow-up questionnaire measures were modified according the participants´ RTW status (see the footnotes in Table 1 for the selected variables).

RTW-related questions included the RTW expectation: “When do you expect to return to your 1) previous company and 2) previous workplace considering your current state of health - within the next 3, 6, 9 or 12 months?”. To assess work ability, the single-item WAS score was used (“current work ability compared with the lifetime best”), with a possible range of 0 (completely unable to work) to 10 (work ability at its best) [41]. A German version of the RTW self-efficacy scale [42] was used, translated by the study team and verified in terms of accuracy through a third party translator. Job crafting (JC) was measured with a modified and shorter version of a German translation [43] and the JC scale [44]. Questions regarding GRTW and RTW−/BEM processes covered the offer, the concrete procedure and satisfaction with it. Employer to employee contact during SA was measured with two questions concerning 1) who has been contacted through the participant and 2) who contacted the participant and one additional question evaluating the helpfulness of each contact on a four-point Likert scale from (1) not helpful at all to (4) very helpful. Helpful (on the four-point Likert scale from above) and necessary work accommodation needs for RTW were questioned at t0 and necessary work accommodation needs for next 2 years were questioned at t3.

Health-related characteristics included the first and second medical diagnoses (ICD-10 codes) through the clinicians. Recurrence of disorder was defined as “no” if the answer to the question “In which year was the diagnosis of your disorder/illness first made?” was 2016 or later and as “yes” if the answer was before the year 2016. Work-relatedness of disorder was measured on a five-point Likert scale from (1) not at all, (2) slightly, (3) moderately, (4) very to (5) extremely. Sickness absence was measured in weeks, at baseline for the last 12 months and at each follow-up for the last 6 months, respectively. Sickness presenteeism was measured with the question “Has it happened over the previous 6 months that you have gone to work despite feeling that you really should have taken sick leave due to your state of health?” with four answering options (1) never, (2) once, (3) two to five times and (4) more than five times [46]. Self-rated health was measured with the question “How would you describe your current health?” on a five-point Likert scale from (1) very good, (2) good, (3) satisfactory, (4) poor to (5) bad. Functional ability was measured with two sub-scales (managing and cooperation/communication) from the German version of the Norwegian Function Assessment Scale [48, 49]. Participants were asked if they have a degree of disability and – if not – whether they want to apply for recognition as a disabled person. Stigma resistance was measured with two-items of the internalized stigma of mental illness (ISMI) scale [55].

#### Primary and secondary outcomes

The primary outcome of the quantitative study is the time until RTW and full RTW (after finishing all GRTW measures) measured in days. The secondary outcome is the sustainability of RTW (SRTW), which often has been defined as ‘28 days of full RTW’ or within longer timeframes of 6 months or 2 years of full RTW [56]. Beyond these definitions solely based on administrative timeframes [cf. 18], in the present study a more comprehensive definition of SRTW will be developed and tested with quantitative and qualitative follow-up data.

#### Data management and statistical analyses

Statistical analyses will be performed with SPSS 24 and Stata 15 (or subsequent versions). Descriptive analysis and interrelationships will be examined. Two types of survival analyses will be conducted. A continuous Kaplan-Meier procedure will be used to analyse the period between the date of discharge of the participant from the clinic or the rehabilitation facility and the first day on the job. In the second survival analysis, cox regression survival models will be conducted to investigate the factors that influence the time to RTW and SRTW. To adjust for clinic and clustering, analyses will be performed using multilevel modelling, where the clinic could be included either as a fixed or random effect. SRTW trajectories will be identified using sequence analysis and subsequently analysed in regression models. In all publications, results will be reported following the STROBE statement (Strengthening the Reporting of Observational Studies in Epidemiology) [57]. In the present paper, we describe quantitative and qualitative baseline findings with a non-response analysis and characteristics of the participants stratified by the two examined mental health services, group differences were analysed with chi-square tests and t-tests (see Table 2).

### Qualitative study

The qualitative data was collected via face-to-face baseline interviews that took place in the clinical setting in the last week before discharge. Both qualitative follow-up interviews after six (t1) and 12 (t2) months were conducted by telephone, and if possible, using the same interviewer from the baseline interview. It was planned to interview 32 participants as a qualitative sub-sample at the first three measurement points. Another eight participants were additionally interviewed at all qualitative measurement points as possible substitutes for losses in the sub-sample. In accordance with receiving the informed consent of participants, all conversations were recorded under their study pseudonym and were anonymised during transcription.

In the three qualitative interviews within a year after treatment (t0-t2), the study participants were asked about their experiences during the RTW process in narrative interviews that lasted on average 45 min. The interviews were transcribed and analysed using the documentary method of interpretation [58]. This method follows the ethnomethodological approach [59, 60]. According to this method, the first step was to reconstruct the implicit experience-based knowledge of the participants concerned in the RTW process. On the basis of their experiences and frameworks for action, abstracted types were reconstructed through case comparisons. Afterwards, a link between the implicit and explicit levels of knowledge was established and a typology was created, which refers to three essential aspects of a RTW process: (1) the path leading to illness or factors associated with the development of the disorder, (2) dealing with the disorder during clinical treatment in the therapeutic setting and finally (3) the process of RTW and its sustainability.

### Baseline findings of the quantitative study

A total of 286 participants were included in the study. After completing the last follow-up in April 2019, the overall response rate 18 months after inclusion was 91% (see Fig. 1 for the study flow).

#### Non-response analysis

Thirteen persons were excluded before the baseline CATI, because they did not meet the inclusion criteria. In addition, seven persons were excluded retrospectively, because their diagnoses (via ICD-10 codes) were not suitable for the study. Eleven persons were already discharged when the study documents reached the project team. Two persons were too ill to participate.

A short anonymous paper-pencil non-response questionnaire about age, sex, RTW expectancies and reasons for non-participation was filled in from 79 persons, who were eligible but refused to participate. No significant differences between participants and non-responders were found regarding age and sex. Non-responders were not as confident as study participants about their RTW expectation: there were significant more non-responders with the intention to RTW without a concrete time perspective (χ^2^ (2, *n* = 349) = 15.56 *p* < .001, *V* = .211). Main reasons for non-participation were (open answer format, answers were categorised by content): *mental overload and too much additional burden* (*n* = 21), *no interest or motivation* (*n* = 16), *data protection issues* regarding diagnosis and safekeeping (*n* = 9) and *the length of the study* (*n* = 7).

#### Characteristics of the participants

Table 2 shows the baseline characteristics for the total cohort as well as stratified by psychiatric and rehabilitation group (due to the afore mentioned differences in the care and funding system). At baseline, participants of the rehabilitation group were on average 3 years older, had more fixed working hours or shift work, less sedentary work, a faster RTW expectation, and reported higher RTW self-efficacy, higher work ability, better self-rated health, greater functional ability and less depressive symptoms than the psychiatric participants. Whereas nearly 36% of the rehabilitation participants were diagnosed with an adjustment disorder as their first diagnosis, all participants of the psychiatric group were diagnosed with a depression or anxiety disorder. The average length of a stay in a psychiatric clinic was more than 2 weeks longer (mean = 7.6 weeks, SD = 1.60) than in a rehabilitation facility (mean = 5.2 weeks, SD = .76; *t* (257) = 16.618, *p* < .001, mean diff. = 2.36, 95% CI [2.08–2.64] η^2^ = .493). Most psychiatric participants received day hospital treatment (76%), whereas almost all rehabilitation participants received inpatient treatment (98%). The median monthly net household income was lower in the rehabilitation group: 2000–3000 Euros vs. 3000–4000 Euros in the psychiatric group (Median Test*, p* < .05). Nearly all participants (97%) had a permanent employment contract.

#### Work accommodation needs for RTW

In Table 3 the ten most important necessary work accommodation needs for RTW from the participants´ perspective are presented. Given a list of 13 possible work accommodations with multiple answers allowed and one free text category, 260 participants reported at least one work accommodation need for their RTW. Gradual RTW was considered the most important necessary work accommodation need for RTW of the psychiatric participants, but significantly less often chosen by the rehabilitation participants, who considered a reduction of workload as most important. Only 26 participants had no work accommodation needs at all, the majority of those (73%) belonged to the rehabilitation group [*Χ*^*2*^ (1, *n* = 286) = 10.82 *p* < .01, *phi* = .207)].

**Table 1:** Overview of the selected measures and measurement points

Measures	Source and reference	Total range	Base line (t0)	6 months (t1)	12 months (t2)	18 months (t3)
Socio-demographic information	Own development and [36]		x			
Work-related						
Job characteristics	Own development and [36, 37]		x			
Quantitative demands	COPSOQ [38]	0 to 100	x		x^a^	x^a^
Influence at work	COPSOQ [38]	0 to 100	x		x^a^	x^a^
Social support (colleagues)	COPSOQ [39]	0 to 100	x	x^a^	x^a^	x^a^
Sense of community	COPSOQ [38]	0 to 100	x	x^a^	x^a^	x^a^
Social support (supervisor)	COPSOQ [39]	0 to 100	x	x^a^	x^a^	x^a^
Quality of leadership	COPSOQ [38]	0 to 100	x	x^a, b^	x^a, b^	x^a, b^
Job insecurity	COPSOQ [38]	0 to 100	x	x^a^	x^a^	x^a^
Work-privacy conflict	COPSOQ [38]	0 to 100	x	x^a^	x^a^	x^a^
Meaning of work	COPSOQ [38]	0 to 100	x			x^a^
Workplace commitment	COPSOQ [38]	0 to 100	x			x^a^
Trust and fairness	COPSOQ [39]	0 to 100	x			x^a^
Overall job satisfaction	COPSOQ [38]	0 to 100	x			x^a^
RTW-related						
RTW expectation	Adapted from SIBAR [40]		x	x^c^	x^c^	x^c^
Work ability	WAS [41]	0 to 10	x	x	x	x
RTW self-efficacy	[42]	1 to 6	x	x	x	x
Job crafting	Adapted from [43, 44]	1 to 5		x^a^	x^a^	x^a^
Current working status	Own development		x	x	x	x
RTW and SRTW trajectories	Own development			x	x	x
GRTW processes	Own development		x	x	x	x
“RTW/BEM” processes	Own development		x	x	x	x
Employer – employee contact during SA	Own development		x	x^c^	x^c^	x^c^
Helpful/necessary work accommodation needs	Own development		x			
Necessary work accommodation needs next 2 years	Own development					x^a^
Health-related						
First & second diagnosis	ICD-10 codes from Clinicians		x			
Recurrence of disorder	Own development		x			
Work-relatedness of disorder	Adapted from Würzburger Screening [45]	1 to 5	x			
Weeks of sickness absence for the last twelve/six months	Own development		x	x	x	x
Sickness presenteeism	[46]		x	x^a^	x^a^	x^a^
Self-rated health	[47]	1 to 5	x	x	x	x
Functional ability	NFAS [48, 49]	1 to 5	x	x	x	x
Depressive symptoms	PHQ-8 [50]	0 to 24	x	x	x	x
Generalized anxiety	GAD-2 [51]	0 to 6	x	x	x	x
Regular physical activity	DEGS [52]		x			
Smoking behaviour	DEGS [52]		x			
Alcohol consumption	Adapted from DEGS [52]		x			
Clinical stay	Adapted from [53]		x			
Comorbidity	Own development		x			
Further treatment	Own development		x	x	x	x
Application for early retirement	Adapted from SPE-Scale [54]			x^c^	x^c^	x^c^
Application for recognition as a disabled person	SIBAR [40]		x			x
Degree of disability	SIBAR [40]		x			x
Stigma resistance	Adapted from ISMI-10 [55]	1 to 4	x	x	x	x

*COPSOQ* Copenhagen Psychosocial Questionnaire, *SIBAR* Screening instrument work and occupation, *WAS* Work ability score, *Würzburger Screening* for Sociomedical Documentation, *PHQ-8* Patient Health Questionnaire Depression Scale-8, *GAD-2* Generalized Anxiety Disorder Scale-2, *DEGS* German Health Interview and Examination Survey for Adults, *ISMI-10* Internalized Stigma of Mental Illness Scale-10. *SPE-Scale* subjective prognosis of gainful employment

^*a*^ only applied if participant did return to work, ^b^ only applied if supervisor changed, ^c^ only applied if participant did not return to work yet

**Table 2:** Baseline characteristics of the total study population and stratified by psychiatric and rehabilitation group

Variables	Total sample (*n* = 286)	Psychiatric group (*n* = 169)	Rehabilitation group (*n* = 117)	Statistics^a^
Sociodemographic				
Sex, % (n)				
Female	46.5 (133)	45.6 (77)	47.9 (56)	*n. s.*
Male	53.5 (153)	54.4 (92)	52.1 (61)	
Age^b^ (years), mean ± SD (n)	47.7 ± 8.6 (286)	46.4 ± 8.7 (169)	49.7 ± 8.2 (117)	*t* (284) = −3.25, *p* < .01
≤ 39 years, % (n)	18.5 (53)	22.5 (38)	12.8 (15)	*Χ*^*2*^ (2, *n* = 286) = 14.95, *p* < .01, *V* = .229
40–49 years, % (n)	29.4 (84)	34.9 (59)	21.4 (25)	
≥ 50 years, % (n)	52.1 (149)	42.6 (72)	65.8 (77)	
Highest educational/ vocational qualification^b^, % (n)				
Qualification from a company/ school-based vocational training	48.3 (138)	45.0 (76)	53.0 (62)	*n. s.*
Qualification from a technical college/ vocational academy	18.5 (53)	16.6 (28)	21.4 (25)	
(Applied) University degree	30.1 (86)	34.9 (59)	23.1 (27)	
Other/ no vocational qualification	3.1 (9)	3.6 (6)	2.6 (3)	
Partnership, % (n)				
No	29.5 (84)	30.2 (51)	28.4 (33)	*n. s.*
Yes	70.5 (201)	69.8 (118)	71.6 (83)	
└ Living Together, % (n)	89.6 (180)	91.5 (108)	86.7 (72)	
Work-related				
Weekly working time, % (n)				
Full-time (≥ 35 h)	79.4 (227)	82.2 (139)	75.2 (88)	*n. s.*
Part-time (15–34 h)	20.6 (59)	17.8 (30)	24.8 (29)	
Working time model^b^, % (n)				
Fixed working hours	25.5 (73)	21.3 (36)	31.6 (37)	*Χ*^*2*^ (2, *n* = 286) = 22.56, *p* < .001, *V* = .281
Flexible working hours	50.7 (145)	62.1 (105)	34.2 (40)	
Shift work	23.8 (68)	16.6 (28)	34.2 (40)	
Mainly sedentary work, % (n)				
No	34.3 (98)	29.6 (50)	41.0 (48)	*Χ*^*2*^ (1, *n* = 277) = 4.07, *p* < .05, *phi* = .129
Yes	62.6 (179)	68.0 (115)	54.7 (64)	
No answer	3.1 (9)	2.4 (4)	4.3 (5)	
Managerial function, % (n)				
No	80.1 (229)	79.3 (134)	81.2 (95)	*n. s.*
Yes	19.9 (57)	20.7 (35)	18.8 (22)	
Enterprise sector, % (n)				
Private	69.6 (199)	72.8 (123)	65.0 (76)	*n. s.*
Public	30.4 (87)	27.2 (46)	35.0 (41)	
Company size, % (n)				
1–50 employees	13.6 (39)	10.7 (18)	17.9 (21)	*n. s.*
51–250 employees	16.8 (48)	14.8 (25)	19.7 (23)	
> 250 employees	69.6 (199)	74.6 (126)	62.4 (73)	
RTW-related				
RTW expectations^b^, % (n)				
≤ 3 months	83.9 (240)	78.1 (132)	92.3 (108)	*Χ*^*2*^ (2, *n* = 286) = 10.33, *p* < .01, *V* = .190
> 3 months	9.1 (26)	12.4 (21)	4.3 (5)	
No return to former workplace	7.0 (20)	9.5 (16)	3.4 (4)	
RTW-SE (1–6), mean ± SD (n)	4.23 ± 1.08 (280)	3.99 ± 1.08 (165)	4.59 ± .99 (115)	*t* (278) = −4.81, *p* < .001
Work ability (0–10), mean ± SD (n)	5.27 ± 2.16 (286)	4.70 ± 2.08 (169)	6.10 ± 2.01 (117)	*t* (284) = −5.69, *p* < .001
Health-related				
First diagnosis^b^, % (n)				
Depression or anxiety disorder	85.3 (244)	100 (169)	64.1 (75)	*Χ*^*2*^ (1, *n* = 286) = 68.27, *p* < .001, *phi* = −.499
Adjustment disorder	14.7 (42)	0	35.9 (42)	
Recurrence of disorder, % (n)				
No	64.0 (183)	72.2 (122)	52.1 (61)	*Χ*^*2*^ (1, *n* = 286) = 11.21, *p* < .01, *phi* = .205
Yes	36.0 (103)	27.8 (47)	47.9 (56)	
Work-relatedness of disorder^b^, % (n)				
Not at all – moderately	33.2 (95)	29.6 (50)	38.5 (45)	*n. s.*
Very – extremely	66.4 (190)	70.4 (119)	60.7 (71)	
No answer	0.3 (1)	0	0.9 (1)	
SA last 12 months^b^ (weeks), mean ± SD (n)	9.96 ± 7.94 (286)	10.86 ± 7.87 (169)	8.65 ± 7.89 (117)	*t* (284) = 2.33, *p* < .05
≤ 6 weeks/12 months, % (n)	44.8 (128)	37.3 (63)	55.6 (65)	*Χ*^*2*^ (1, *n* = 286) = 8.62, *p* < .01, *phi* = −.181
> 6 weeks/12 months, % (n)	55.2 (158)	62.7 (106)	44.4 (52)	
Self-rated health^b^, % (n)				
Good	75.9 (217)	71.6 (121)	82.1 (96)	*Χ*^*2*^ (1, *n* = 286) = 3.58, *p* < .05, *phi* = .120
Poor	24.1 (69)	28.4 (48)	17.9 (21)	
Functional ability (1–5), mean ± SD (n)				
Managing	2.31 ± .79 (249)	2.45 ± .78 (154)	2.08 ± .77 (95)	*t* (247) = 3.66, *p* < .001
Cooperation/Communication	1.89 ± .75 (281)	2.04 ± .79 (166)	1.69 ± .64 (115)	*t* (273) = 4.13, *p* < .001
Depressive symptoms (0–24), mean ± SD (n)	7.79 ± 4.58 (282)	8.31 ± 4.54 (167)	7.03 ± 4.56 (115)	*t* (280) = 2.33, *p* < .05
Regular physical activity, % (n)				
< 2.5 h/week	65.4 (187)	64.5 (109)	66.7 (78)	*n. s.*
≥ 2.5 h/week	34.3 (98)	34.9 (59)	33.3 (39)	
No answer	0.3 (1)	0.6 (1)	0	
Smoking behaviour, % (n)				
Current smoker (daily/occasional)	30.1 (86)	29.6 (50)	30.8 (36)	*n. s.*
Ex-smoker	36.7 (105)	39.1 (66)	33.3 (39)	
Non-smoker	33.2 (95)	31.4 (53)	35.9 (42)	
Alcohol consumption^b^, % (n)				
Never or once/month	35.0 (100)	37.3 (63)	31.6 (37)	*Χ*^*2*^ (2, *n* = 286) = 11.45, *p* < .01, *V* = .200
2–4 times/month	42.0 (120)	34.3 (58)	53.0 (62)	
≥ 2 times/week	23.1 (66)	28.4 (48)	15.4 (18)	

^a^ To test for significant relationships and differences between the groups, either chi-square tests for independence (where required with Yates´ Continuity Correction) or independent-samples t-tests were conducted. ^b^ To ensure better reading and/or participant privacy, the categories of this variable were aggregated

**Table 3:** Ten most important necessary work accommodation needs for RTW

Nr	Work accommodation need	Total sample (n = 286), %, (n)	Psychiatric group (*n* = 169), %, (n)	Rehabilitation group (*n* = 117), %, (n)	Statistics
1	GRTW	48.6 (139)	65.7 (111)	23.9 (28)	*Χ*^*2*^ (1, *n* = 286) = 46.58, *p* < .001, *phi* = −.411
2	Reduction of workload	31.8 (91)	33.7 (57)	29.1 (34)	n. s.
3	Regular feedback talks with supervisor	25.2 (72)	29.0 (49)	19.7 (23)	n. s.
4	Improvement of work organisation	24.1 (69)	26.6 (45)	20.5 (24)	n. s.
5	Reduction of working time	18.9 (54)	24.3 (41)	11.1 (13)	*Χ*^*2*^ (1, *n* = 286) = 6.97, *p* < .01, *phi* = −.158
6	Training event for colleagues & supervisors in dealing with mental disorders	17.5 (50)	19.5 (33)	14.5 (17)	n. s.
7	Individual RTW-support	13.3 (38)	17.8 (30)	6.8 (8)	*Χ*^*2*^ (1, *n* = 286) = 6.23, *p* < .05, *phi* = −.165
8	Flexibility of working times	11.5 (33)	11.8 (20)	11.1 (13)	n. s.
9	Improvement of working environment	10.1 (29)	8.3 (14)	12.8 (15)	n. s.
10	Change of workplace	10.1 (29)	11.8 (20)	7.7 (9)	n. s.

Note: Multiple answers were allowed

### Baseline findings of the qualitative study

#### Qualitative sub-sample

A total of 95 interviews (out of 96 planned interviews) were conducted. One participant dropped-out after t0 and was substituted by another participant for the additional interviews, see Fig. 1. One interview could not be performed at t2, but it was decided that the first two interviews were sufficient for the analysis and the case was not replaced. No differences regarding the baseline characteristics were found for the qualitative sub-sample (*n* = 32) compared to the quantitative sample, except for working in private sector enterprises: 47% of the qualitative sample vs. 70% of the quantitative sample [(*Χ*^*2*^ (1, *n* = 286) = 7610 *p* < .05, *phi* = .175)] did so.

#### Paths into crisis/illness

The following main causes for the emergence of the disorder were described by the participants: (1) excessive demands caused by working conditions, attitudes towards work and conflicts at work (46.9%, *n* = 15); (2) excessive demands caused by individual factors and biographical circumstances (15.6%, *n* = 5); (3) a combination of 1 and 2 (37.5%, *n* = 12). For the interviewed participants work-related factors played an important role when dealing and coping with the disorder.

#### RTW expectations

Coping with the disorder and the further RTW process seemed to be especially problematic for participants with a negative RTW expectation (> 3 months) and those, whose crisis was caused by a complex combination of work-related and personal factors: stress at work, high willingness to exert oneself, low willingness to set boundaries, personal problems (e.g. caring for a family member and/or problems with one’s partner) and a biographical burden (e.g. history of abuse in the family).

#### RTW facilitators

Four important factors seemed to have a positive effect on the course of RTW: (1) how open and active the participants were when dealing with their situation or disorder during the clinical stay, (2) how they developed coping strategies for their further RTW process, (3) how they assessed their employer’s support and (4) how concrete their RTW expectations were.

## Discussion

This paper provides an overview of the study design, recruitment, sample characteristics including a non-response analysis and baseline findings of the first (S)RTW mixed-methods follow-up cohort study in Germany. A total of 286 participants were included in the cohort with an overall response rate of 91% completing all four telephone surveys. Comparing the qualitative sub-sample of 32 participants with the quantitative cohort revealed only one minor difference in the baseline characteristics. The study cohort consists of two German mental health services sub-groups, a psychiatric and a rehabilitation group, which as expected (due to afore mentioned psychiatric and rehabilitation criteria) differ in many baseline characteristics and therefore should be considered in ongoing data analysis.

The quantitative study shows that the vast majority of the participants had a positive expectation to RTW within 3 months, with the participants from the rehabilitation facilities being more optimistic and expecting a faster return than the psychiatric participants. This result is in line with the better health situation reported by the rehabilitation group. Nevertheless, their reported lower monthly household income could also be a reason for their faster RTW expectation and should be further analysed. In addition, the rehabilitation participants chose less work accommodation needs for their RTW than the psychiatric participants, especially GRTW and reduced working hours were less often considered necessary in the rehabilitation group. Again, the financial situation together with their less flexible working situation should be further analysed, because, as mentioned before, during GRTW employees receive sickness absence benefits at about 70% of the full salary. It is possible that rehabilitation participants may choose GRTW more infrequently because of their lower income and an inability to compensate the financial cost imposed by this work accommodation. For participants with a slower (negative) RTW expectation (> 3 months), whose crisis aroused from a complex combination of work-related and personal factors, the qualitative study found that this group has special needs in terms of disease coping and individual support for RTW. As a result, subsequent analyses are planned on the quantitative and qualitative follow-up data.

Despite the overall response rate, which provides a very good basis for further analyses, some limitations have to be kept in mind. Because the present study was designed as an observational prospective cohort study without a control group, no control over other confounding variables is possible. In Germany, no register data on sickness absence periods are centrally available, so that we had to use self-reported data except for the medical first and second diagnoses from the clinicians. Thereby, the differences in the distribution of the given diagnoses between the two groups could be possibly explained by the different systems and their access paths, care routines, diagnostic procedures and the fact, that patients with adjustment disorders have commonly a shorter duration in the acute psychiatric setting (of the selected cooperation clinics). For this reason, we were not able to recruit study participants with adjustment disorders in the psychiatric setting (as an example see distributions of adjustment disorders of different inpatient units in [61]). As the selection of participants was undertaken with cooperating clinics for reasons of recruitment, the study sample is not representative for patients with a CMD in Germany as well as for the two examined mental health service groups. Furthermore a non-probabilistic sample has been drawn, which should be kept in mind for further analyses. Due to the narrow study inclusion criteria, a healthy entrance effect may have occurred. Finally, as the study was designed from the perspective of the employees, the perspective of the employers and key RTW stakeholders is missing.

To the best of our knowledge, this study is the first of its kind to investigate RTW and SRTW processes due to CMDs in Germany. It addresses an important research gap. One further advantage of this study is the use of well-validated instruments that have previously shown high internal consistency and strong validity. Moreover, the long term observation period of 18 months combined with a mixed method approach will add important knowledge on RTW and SRTW processes over time. As a result, recommendations for employers and other stakeholders on promoting and supporting (S)RTW practices in Germany can be given. Based on that knowledge, interventions can be developed to improve (S)RTW processes and practices in Germany in the future.

## Data Availability

The datasets analysed during the current study are available from the corresponding author on reasonable request after official permission of the privacy officer from the Federal Institute for Occupational Safety and Health.

## Abbreviations

BEM: “Betriebliches Eingliederungsmanagement” (operational reintegration management)
CMD: Common Mental Disorder
COPSOQ: Copenhagen Psychosocial Questionnaire
GRTW: Gradual Return to Work
JC: Job Crafting
RTW: Return to Work
SA: Sickness Absence
SRTW: Sustainable Return to Work

## References

[1] Jacobi F, Hofler M, Siegert J, Mack S, Gerschler A, Scholl L (2014). Twelve-month prevalence, comorbidity and correlates of mental disorders in Germany: the mental health module of the German health interview and examination survey for adults (DEGS1-MH). Int J Methods Psychiatr Res.

[2] Jacobi F, Hofler M, Strehle J, Mack S, Gerschler A, Scholl L (2015). Twelve-months prevalence of mental disorders in the German health interview and examination survey for adults - mental health module (DEGS1-MH): a methodological addendum and correction. Int J Methods Psychiatr Res.

[3] BMAS/BAuA (2018). Sicherheit und Gesundheit bei der Arbeit – Berichtsjahr 2017.

[4] OECD (2015). Mental health and work: fit mind, fit job: from evidence to practice in mental health and work.

[5] Henderson M, Harvey SB, Overland S, Mykletun A, Hotopf M (2011). Work and common psychiatric disorders. J R Soc Med.

[6] Wittchen HU, Jacobi F, Rehm J, Gustavsson A, Svensson M, Jonsson B (2011). The size and burden of mental disorders and other disorders of the brain in Europe 2010. Eur Neuropsychopharmacol.

[7] van Rijn RM, Robroek SJ, Brouwer S, Burdorf A (2014). Influence of poor health on exit from paid employment: a systematic review. Occup Environ Med.

[8] Mäcken J (2019). Work stress among older employees in Germany: effects on health and retirement age. PLoS One.

[9] Hiilamo A, Shiri R, Kouvonen A, Mänty M, Butterworth P, Pietiläinen O (2019). Common mental disorders and trajectories of work disability among midlife public sector employees – a 10-year follow-up study. J Affect Disord.

[10] Wedegaertner F, Arnhold-Kerri S, Sittaro N, Bleich S, Geyer S, Lee WE. Depression- and anxiety-related sick leave and the risk of permanent disability and mortality in the working population in Germany: a cohort study. BMC Public Health. 2013;13:145. 10.1186/1471-2458-13-145 PMC3698165 23413800

[11] Mack S, Jacobi F, Gerschler A, Strehle J, Hofler M, Busch MA (2014). Self-reported utilization of mental services in the adult German population – evidence for unmet needs? Results of the DEGS1-mental health module (DEGS1-MH). Int J Methods Psychiatr Res.

[12] Salize HJ, Roessler W, Becker T (2007). Mental health care in Germany: current state and trends. Eur Arch Psychiatry Clin Neurosci.

[13] Gaebel W, Kowitz S, Zielasek J (2012). The DGPPN research project on mental healthcare utilization in Germany: inpatient and outpatient treatment of persons with depression by different disciplines. Eur Arch Psychiatry Clin Neurosci.

[14] de Vries H, Fishta A, Weikert B, Rodriguez Sanchez A, Wegewitz U (2018). Determinants of sickness absence and return to work among employees with common mental disorders: a scoping review. J Occup Rehabil.

[15] Nigatu YT, Liu Y, Uppal M, McKinney S, Gillis K, Rao S (2017). Prognostic factors for return to work of employees with common mental disorders: a meta-analysis of cohort studies. Soc Psychiatry Psychiatr Epidemiol.

[16] Ervasti J, Joensuu M, Pentti J, Oksanen T, Ahola K, Vahtera J (2017). Prognostic factors for return to work after depression-related work disability: a systematic review and meta-analysis. J Psychiatr Res.

[17] Andersen MF, Nielsen KM, Brinkmann S (2012). Meta-synthesis of qualitative research on return to work among employees with common mental disorders. Scand J Work Environ Health.

[18] Nielsen K, Yarker J, Munir F, Bueltmann U (2018). IGLOO: an integrated framework for sustainable return to work in workers with common mental disorders. Work Stress.

[19] Lammerts L, Schaafsma FG, Bonefaas-Groenewoud K, van Mechelen W, Anema J (2016). Effectiveness of a return-to-work program for workers without an employment contract, sick-listed due to common mental disorders. Scand J Work Environ Health.

[20] Young Amanda E., Roessler Richard T., Wasiak Radoslaw, McPherson Kathryn M., van Poppel Mireille N. M., Anema J. R. (2005). A Developmental Conceptualization of Return to Work. Journal of Occupational Rehabilitation.

[21] Etuknwa Abasiama, Daniels Kevin, Eib Constanze (2019). Sustainable Return to Work: A Systematic Review Focusing on Personal and Social Factors. Journal of Occupational Rehabilitation.

[22] McDowell C, Fossey E (2015). Workplace accommodations for people with mental illness: a scoping review. J Occup Rehabil.

[23] Aufwendungsausgleichsgesetz - AAG https://www.gesetze-im-internet.de/aufag/ Accessed on 28 May 2019.

[24] Entgeltfortzahlungsgesetz - EntgFG https://www.gesetze-im-internet.de/entgfg/ Accessed on 28 May 2019.

[25] Streibelt M, Buerger W, Nieuwenhuijsen K, Bethge M (2018). Effectiveness of graded return to work after multimodal rehabilitation in patients with mental disorders: a propensity score analysis. J Occup Rehabil.

[26] Stegmann R, Schroeder U (2016). Psychische Erkrankungen in der Arbeitswelt: Wiedereingliederung nach einer psychischen Krise. ASU Arbeitsmed Sozialmed Umweltmed.

[27] Schneider U, Linder R, Verheyen F (2016). Long-term sick leave and the impact of a graded return-to-work program. Eur J Health Econ.

[28] Bönisch S, Ernst R. Medizinische Rehabilitation. In: Bundesarbeitsgemeinschaft für Rehabilitation e.V. (BAR), editor. Rehabilitation: Vom Antrag bis zur Nachsorge – für Ärzte, Psychologische Psychotherapeuten und andere Gesundheitsberufe. Berlin Heidelberg: Springer-Verlag; 2018.

[29] Creswell JW, Clark VLP. Designing and Conducting Mixed Methods Research. Los Angeles: SAGE Publications; 2018.

[30] Gaebel W, Kowitz S, Fritze J, Zielasek J (2013). Use of health care services by people with mental illness. Dtsch Arztebl Int.

[31] Hendlmeier I, Hoell A, Schäufele M (2015). Kompendium: Leitfaden Psychische Problemlagen.

[32] Deutsche Rentenversicherung Bund (2015). Positionspapier der Deutschen Rentenversicherung zur Bedeutung psychischer Erkrankungen in der Rehabilitation und bei Erwerbsminderung.

[33] Bethge M, Mattukat K, Fauser D, Mau W (2017). Rehabilitation access and effectiveness for persons with back pain: the protocol of a cohort study (REHAB-BP, DRKS00011554). BMC Public Health.

[34] Zobel A, Meyer A. Psyche & Psychosomatik. In: Bundesarbeitsgemeinschaft für Rehabilitation e.V. (BAR), editor. Rehabilitation: Vom Antrag bis zur Nachsorge – für Ärzte, Psychologische Psychotherapeuten und andere Gesundheitsberufe. Berlin Heidelberg: Springer-Verlag; 2018.

[35] Munz D, Worringen U, Clever U. Psychotherapeuten. In: Bundesarbeitsgemeinschaft für Rehabilitation e.V. (BAR), editor. Rehabilitation: Vom Antrag bis zur Nachsorge – für Ärzte, Psychologische Psychotherapeuten und andere Gesundheitsberufe. Berlin: Springer-Verlag; 2018.

[36] Rose U, Friedland I, Pattloch D (2017). FDZ-Datenreport 5/2017: Studie Mentale Gesundheit bei der Arbeit (S-MGA).

[37] BIBB/BAuA (2012). BIBB/BAuA-Erwerbstätigenbefragung 2011/2012: Arbeit und Beruf im Wandel, Erwerb und Verwertung beruflicher Qualifikationen: BIBB/BAuA.

[38] Nübling M, Stößel U, Hasselhorn HM, Michaelis M, Hofmann F (2005). Methoden zur Erfassung psychischer Belastungen: Erprobung eines Messinstrumentes (COPSOQ).

[39] Pejtersen JH, Kristensen TS, Borg V, Bjorner JB (2010). The second version of the Copenhagen psychosocial questionnaire. Scand J Public Health.

[40] Bürger W, Deck R, Raspe H, Koch U. Screening-Instrument Beruf und Arbeit in der Rehabilitation: Entwicklung und Implementierungsmöglichkeiten eines generischen Screening-Instrumentes zur Identifikation von beruflichen Problemlagen und des Bedarfes an berufsorientierten und beruflichen Rehabilitationsleistungen; 2007.

[41] Ahlstrom L, Grimby-Ekman A, Hagberg M, Dellve L (2010). The work ability index and single-item question: associations with sick leave, symptoms, and health – a prospective study of women on long-term sick leave. Scand J Work Environ Health.

[42] Lagerveld SE, Blonk RWB, Brenninkmeijer V, Schaufeli WB (2010). Return to work among employees with mental health problems: development and validation of a self-efficacy questionnaire. Work Stress.

[43] Vogt K, Hakanen JJ, Brauchli R, Jenny GJ, Bauer GF (2016). The consequences of job crafting: a three-wave study. Eur J Work Organ Psy.

[44] Petrou P, Demerouti E, Peeters MCW, Schaufeli WB, Hetland J (2012). Crafting a job on a daily basis: contextual correlates and the link to work engagement. J Organ Behav.

[45] Löffler S, Wolf HD, Gerlich C, Vogel H (2008). Benutzermanual für das Würzburger Screening: Universität Würzburg.

[46] Aronsson G (2000). Sick but yet at work. An empirical study of sickness presenteeism. J Epidemiol Community Health.

[47] TNS Infratest Sozialforschung (2015). SOEP Survey Papers 419: SOEP-Core – 2015: Personenfragebogen (mit Verweis auf Variablen).

[48] Jankowiak S, Rose U, Kersten N (2018). Application of the ICF based Norwegian function assessment scale to employees in Germany. J Occup Med Toxicol.

[49] Østerås N, Brage S, Garratt A, Benth JS, Natvig B, Gulbrandsen P (2007). Functional ability in a population: normative survey data and reliability for the ICF based Norwegian function assessment scale. BMC Public Health.

[50] Kroenke Kurt, Spitzer Robert L (2002). The PHQ-9: A New Depression Diagnostic and Severity Measure. Psychiatric Annals.

[51] Kroenke K, Spitzer RL, Williams JB, Monahan PO, Löwe B (2007). Anxiety disorders in primary care: prevalence, impairment, comorbidity, and detection. Ann Intern Med.

[52] Robert Koch-Institut (2009). Gesundheitsfragebogen 18 bis 64 Jahre. DEGS Studie zur Gesundheit Erwachsener in Deutschland.

[53] Wienert J, Bethge M. Index berufliche Orientierung. Available from: https://www.thieme-connect.de/media/rehabilitation/EFirst/supmat/10-1055-a-0604-0157-481-0001.pdf Accessed on 28 May 2019.

[54] Mittag O, Glaser-Möller N, Ekkernkamp M, Matthis C, Heon-Klin V, Raspe A (2003). Prädiktive Validität einer kurzen Skala zur subjektiven Prognose der Erwerbstätigkeit (SPE-Skala) in einer Kohorte von LVA-Versicherten mit schweren Rückenschmerzen oder funktionellen Beschwerden der inneren Medizin. Soz Praventivmed.

[55] Boyd JE, Otilingam PG, DeForge BR (2014). Brief version of the internalized stigma of mental illness (ISMI) scale: psychometric properties and relationship to depression, self-esteem, recovery orientation, empowerment, and perceived devaluation and discrimination. Psychiatr Rehabil J.

[56] Young AE, Viikari-Juntura E, Boot CRL, Chan C, Ruiz de Porras DG, Linton SJ (2016). J Occup Rehabil.

[57] von Elm E, Altman DG, Egger M, Pocock SJ, Gotzsche PC, Vandenbroucke JP (2008). The strengthening the reporting of observational studies in epidemiology (STROBE) statement: guidelines for reporting of observational studies. Internist (Berl).

[58] Bohnsack R (2010). Rekonstruktive Sozialforschung: Einführung in qualitative Methoden.

[59] Garfinkel H (1967). Studies in ethnomethodology.

[60] Mannheim K, Kettler D, Meja V, Stehr N (1980). Eine soziologische Theorie der Kultur und ihrer Erkennbarkeit (Konjunktives und kommunikatives Denken). Strukturen des Denkens.

[61] Bichescu-Burian D, Cerisier C, Czekaj A, Grempler J, Hund S, Jaeger S (2017). Patienten mit Störungen nach ICD-10 F3 und F4 in Psychiatrie und Psychosomatik – wer wird wo behandelt? Merkmale der Zuweisung aus der PfAD-Studie. Nervenarzt..

